# Understanding Interphases and Interfaces of Battery Materials at the Nanoscale

**DOI:** 10.1002/smll.202504379

**Published:** 2025-07-17

**Authors:** Sergio Federico Mayer, Benjamin Mercier‐Guyon, Célia Doublet, Adrien Fauchier‐Magnan, Léa Rose Mangani, Corentin Renais, Magda Reuter, Oskar Thompson, Lucas Trassart, Claire Villevieille

**Affiliations:** ^1^ Univ. Grenoble Alpes, Univ. Savoie Mont Blanc, CNRS Grenoble INP, LEPMI Grenoble 38000 France; ^2^ Univ. Grenoble Alpes, CEA, Liten, DEHT Grenoble 38000 France; ^3^ Arkema–Centre de Recherche Rhône‐Alpes Rue Henri Moissan–CS 42063 Pierre Benite Cedex 69491 France

**Keywords:** beam damage, buried interface, cathode‐electrolyte interphase, interphase, lithium‐ion batteries, native layer, solid‐electrolyte interphase

## Abstract

Battery performance and longevity are critically dependent on interfacial characteristics, regardless of whether these interfaces are organic, inorganic, or buried. Comprehensive understanding of these regions is essential for optimizing electrochemical performance. Characterization of battery interfaces presents significant challenges due to their nanometer‐scale thickness, complex composition (often a mixture of organic and inorganic decomposition products), and susceptibility to environmental factors and beam damage. In situ and *operando* techniques, often utilizing synchrotron or neutron sources, are preferred to minimize contamination and capture dynamic interfacial evolution. However, experimental constraints limit universal applicability; vacuum‐based methods suitable for solid‐state batteries are incompatible with liquid electrolyte systems, while buried interfaces pose unique analytical hurdles. A lack of standardized characterization protocols contributes to data variability and potential bias within literature. This review addresses strategies for investigating buried interfaces and examines advanced characterization techniques commonly employed in lithium‐ion battery interface studies. This study specifically addresses concerns surrounding data interpretation and the inherent sensitivity of these layers, highlighting the need for careful methodological consideration and rigorous data validation to ensure accurate representation of interfacial behavior.

## Graphical abstract

Decoding Battery Interfaces. This review examines the significant challenges in characterizing battery interfaces—thin, complex layers vital for performance. Advanced techniques are surveyed, including in situ methods, and discuss data interpretation pitfalls. The complexity of these buried layers is stressed and highlights the need for standardized approaches to advance battery technology.

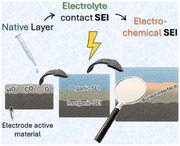

## 1 Introduction

“Harder, better, faster, stronger” seems to be the motto when it comes to battery development.^[^ ^1^ ^]^ Despite an unchanged working principle since the one introduced by Alessandro Volta, the scientific community is perpetually on a quest to develop novel electroactive materials and chemistries that could provide higher energy, higher power density, and enhanced safety, a few of the requirements for innovative electrochemical systems.^[^ ^2^, ^3^ ^]^ Batteries, particularly Li‐ion batteries, are being imposed as a solution that must be constantly improved to reach new goals, such as reliable E‐mobility.

When it comes to developing novel electrochemical systems, one goal is to understand the side reaction mechanisms occurring before, during, and after cycling. For this, researchers and companies rely on multiscale characterization techniques that provide information at various spatial resolutions, from millimeters to nanometers, and try to correlate the degradation arising from the bulk, surface, and interfaces with battery performance.^[^ ^4^, ^5^ ^]^ Understanding the evolution of interfaces and their properties during cycling is of utmost importance, as it is the main reason behind battery aging. However, their proper investigation is hindered not only by their sensitivity but also by their hidden nature, as in the case of the so‐called “buried” interfaces.^[^ ^6^, ^7^ ^]^ Furthermore, the lack of a common methodology in the literature leads to scattered results, even for similar battery systems and characterization methods, making it difficult to advance toward the energy transition.

Revealing the nature of these interfaces/interphases is a critical step toward overcoming current battery limitations and advancing sustainable energy technologies. In the scientific discussion, the following questions were raised:

- Can the sample preparation preserve the integrity of the interface layer?
- Given the highly sensitive nature of this layer, can we rely on the collected data?
- Can we compare the results between different authors?

Here, we present an overview of the techniques employed in the field of interface characterization when organic or inorganic electrolytes are employed. Subsequently, we will discuss limitations in current techniques when it comes to interfacial investigations, including beam damage, native surface layers, and the lack of common methodology.

### 1.1 What is a “Buried Interface”?

The concept of buried interfaces was first introduced in semiconductor materials field^[^ ^8^ ^]^ in 1984. Shortly after, the concept was taken to describe hidden interfaces in the field of batteries.^[^ ^9^ ^]^ You and Nagy described a buried interface as a portion of solid electrode surface covered by a relatively thick layer of liquid electrolyte, with the natural consequence of being hard to access for characterization.

**Scheme 1:**
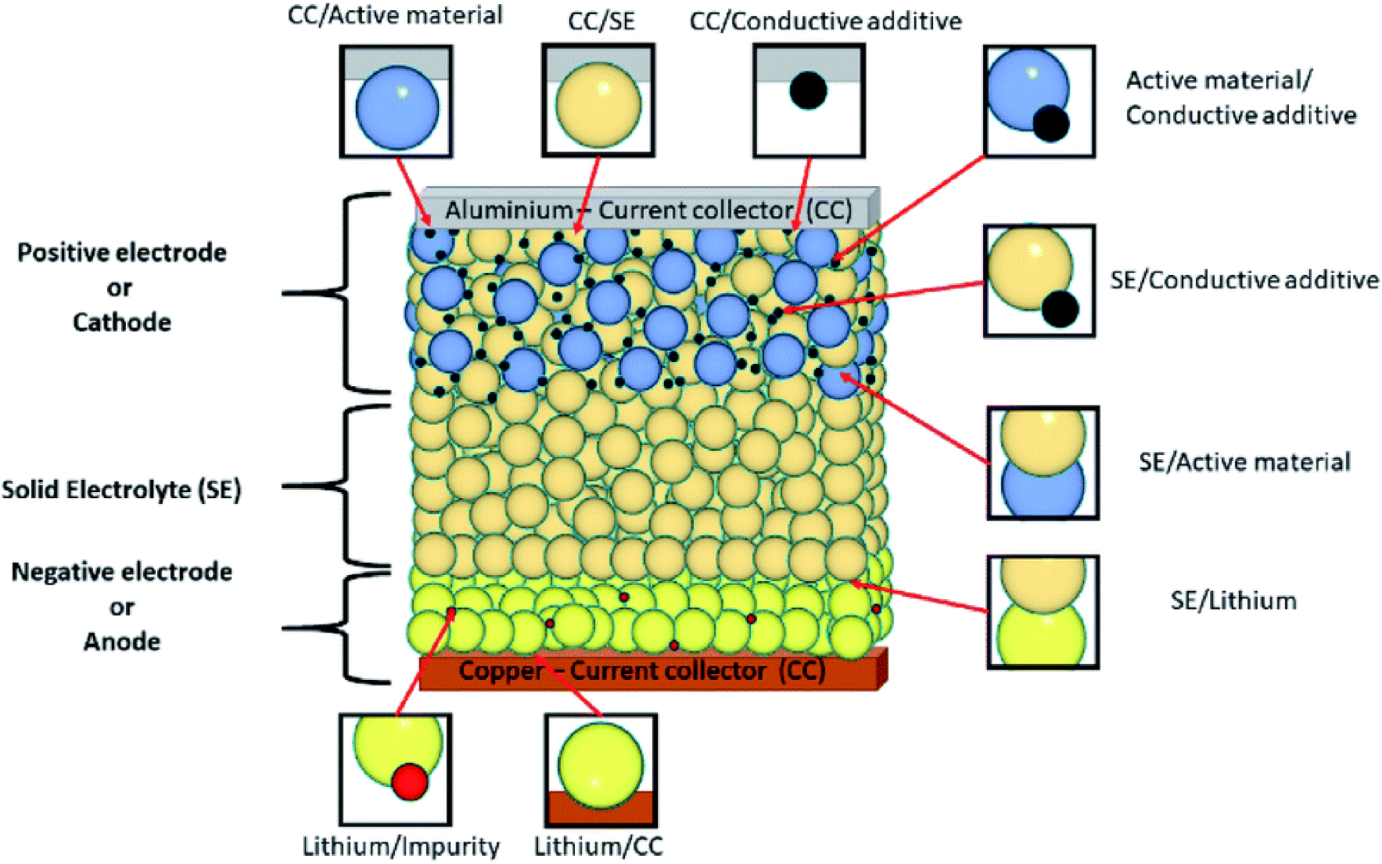
Schematic illustration of buried interfaces present in a solid‐state battery with a metallic negative electrode. The scheme highlights all nine pairwise contacts between typical solid‐state battery components. Used with permission of the Royal Society of Chemistry, from ref. [6]; permission conveyed through Copyright Clearance Center, Inc.

With the growing interest in more complex battery systems, such as solid‐state and hybrid batteries, the definition of buried interface also evolved. In a typical solid‐state battery, nine distinct buried interfaces can form when considering all pairwise contacts between the cell components.^[^ ^6^ ^]^ Each of these interfaces, represented in **Scheme** **1**, is buried between the adjacent materials and cannot be easily accessed: separating them would lead to changes on the structure; microstructure, and chemical composition, losing relevant interface information.^[^ ^6^, ^10^ ^]^ With this initial report, a buried interface could be defined as follows:

*“The interfacial region that is **physically enclosed within a material system**, typically between a solid and at least one other phase (solid or liquid) and is therefore **inaccessible without disrupting** the structural or chemical **integrity of the system**. It is **physically inaccessibility**, as it cannot be exposed without being altered, its **characterization is challenging** as conventional ex situ techniques often fail to capture buried interface properties without artifacts, and it is **important during battery operation,** as these interfaces often dominate processes such as ion transfer, leading to interfacial degradation, and resistance build‐up”*.

### 1.2 Why is Understanding Interfacial Reactivities Important for the Field of Batteries?

Investigation of interfaces, including buried ones, is crucial to having a better understanding of their formation mechanisms, their role in electrochemical processes (e.g., aging), and their interactions with the other battery material.^[^ ^11^ ^]^ To date, it remains unclear whether limiting factors in battery operation are primarily related to transport properties, transfer properties, or a combination of both. However, interfacial behavior likely plays a significant role in both. Interface investigation is challenging because of the complexity of this layer. Typically, it is extremely thin (couple of nanometers), sensitive, and has a complex architecture (usually composed of several sublayers). Furthermore, these layers are potentially “hidden” below the interfaces. Recent advanced characterization techniques have spurred a renewed interest in buried interfaces.

These hidden layers are believed to hold crucial information about the limitations of battery performance, yet their direct probing remains an ongoing challenge. Attempts were made to overcome these challenges by using different techniques such as transmission electron diffraction measurements, extended X‐ray absorption fine‐structure characterization, neutron reflectivity, and synchrotron surface X‐ray scattering studies. Although the initial results were promising, the inherent difficulty of their analysis prompted a shift in focus toward the solid electrolyte interphase (*SEI*) and other passivation layers, which are still debated in the literature.^[^ ^12^ ^]^ The main goal was to reveal the chemical composition, structure, microstructure, and topography^[^ ^13^ ^]^ of the SEI to ultimately establish a direct correlation between a stable SEI and proper capacity retention in Li‐ion batteries. Researchers are constantly pushing the boundaries, seeking novel approaches to reveal these hidden layers without compromising their integrity, but current methodologies still leave room for uncertainty. With advances in the development of novel electrochemical systems, such as solid‐state batteries, polymer‐based batteries, and aqueous systems, interest in these novel interfaces is growing. This is because novel reaction types may be uncovered, and the progress of innovative characterization techniques could facilitate the revaluation of well‐known SEI processes with greater accuracy. Progress in solid‐state batteries and polymer‐based batteries has emphasized the critical role of interfaces because lithium‐ion transport occurs through an interface that is typically more resistive than in liquid systems, hence relying on optimal contact between the solid electrolyte and the electroactive materials. The inability to directly assess buried interfaces raises critical questions about the reliability of data collected through surface investigations and whether such techniques accurately reflect the real operation of a battery. This emphasizes the need for a more thorough, but especially adequate, investigation of surface‐related mechanisms, tailored to the specific battery system being studied, whether it uses a liquid or solid electrolyte, whether the potential decomposition products are organic or inorganic, and how they are structured and located at the interfaces of the various battery components.

**Figure 1:**
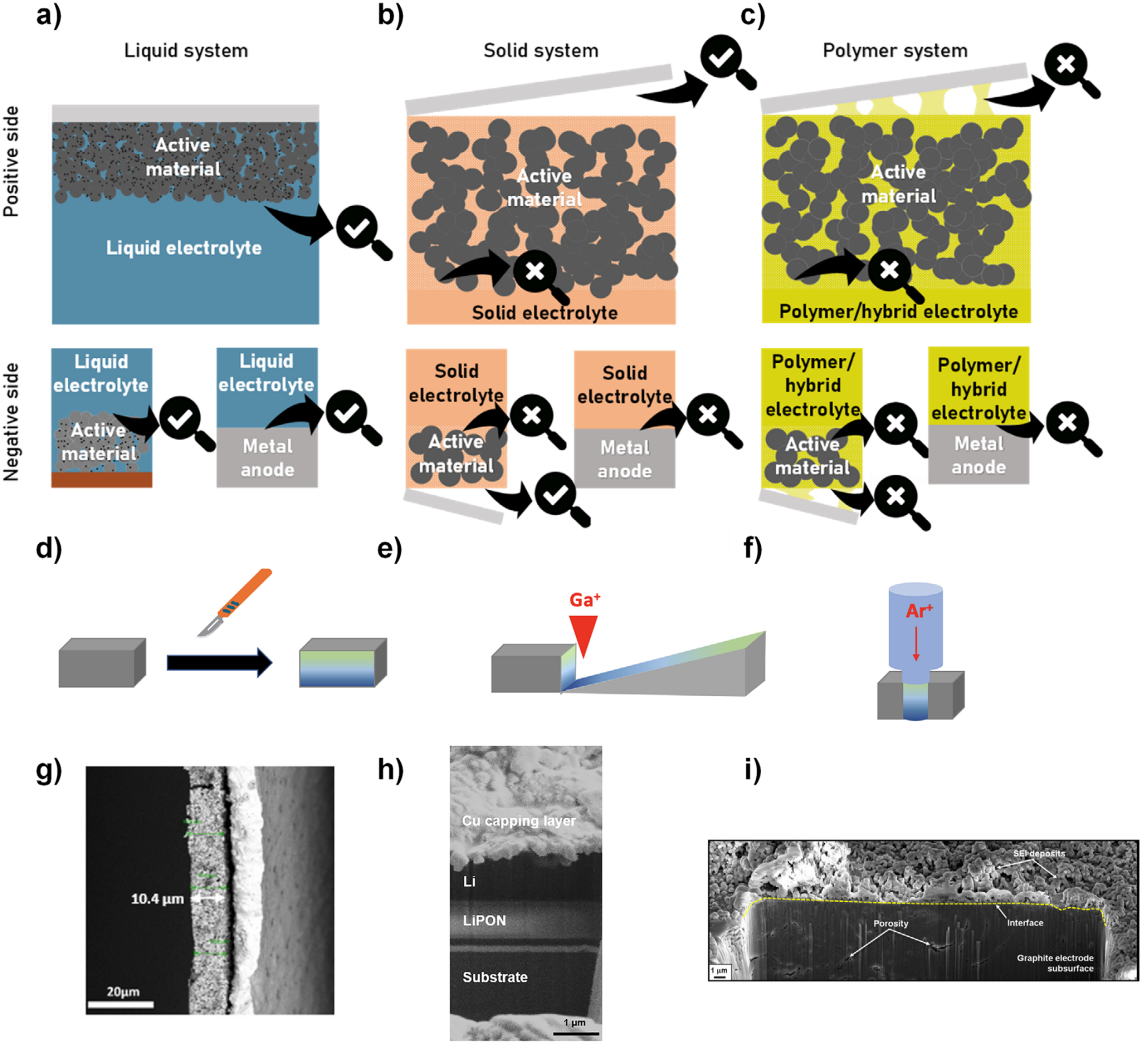
Scheme representing liquid, solid, and polymer/hybrid cell disassembly. This scheme is based on the type of electrolyte employed and illustrates different sample preparation methods for characterization. a) Battery system using a liquid electrolyte. Both interfaces can be easily investigated by dismantling the cell and separating the components. b) Solid state system using a solid electrolyte. The current collector can be removed for direct measurement at the active material‐electrolyte interface, but investigating the interface between the Li metal and the solid electrolyte presents challenges. c) Polymer/hybrid system where the adhesive nature of the polymer electrolyte makes difficult the removal of the current collector and the investigation of the electrode/electrolyte interfaces. To access this area of interest, researchers have proposed various techniques, as demonstrated in (d–f) and exemplified by (g,h,i). d) mechanical slicing along the cross‐section, enabling the exploration of depth profiles as can be seen in (g) Cross‐sectional SEM images of the silicon electrodes with (a) FP2SA binder. Reprinted from ref. [16]. Copyright (2019) American Chemical Society. e) Ablation of the upper layers performed by Focused Ion Beam (FIB) techniques either at room temperature or in cryogenic mode, exemplified by (h) Cryo‐FIB‐SEM cross‐sectional image of the Li/LiPON sample. Adapted from ref. [17] and f) ion milling techniques, frequently coupled with chemical analysis of sample fragment as demonstrated in (i) FIB‐milled cross‐sectional microstructure of a graphite electrode cycled from V = 3.00 → 0.02 V at a low scan rate of 0.05 mV s^−1^. Adapted from ref. [17].

**Figure** **1** illustrates the three main battery systems: liquid electrolyte, solid electrolyte, and polymer‐based electrolyte (Figures 1a–c, respectively). When investigating the interfaces within these systems, it is important to consider the specific chemistry of the battery, as each configuration presents unique challenges and limitations.

#### 1.2.1 Liquid‐Based System: Clear Interface Between the Liquid and Solid Phase

Liquid electrolyte systems (Figure 1a) are relatively easy to disassemble due to the clear separation of the liquid and solid phases. The separator, typically a rigid polymer like Celgard or a glass fiber, can be easily removed, allowing access to the interphases at the surface of the composite electrodes and/or the metal negative electrode. This system facilitates the investigation of interfaces such as SEI or Li metal, as they can be readily exposed. Ex situ analyses are particularly convenient because researchers can readily investigate samples using various characterization techniques. However, washing and drying of the samples are often necessary, potentially altering the composition and structure of the sensitive *SEI* and cathode‐electrolyte interphase (*CEI*) layers. These alterations introduce uncertainty into the analysis, as even minor compositional changes during sample preparation can significantly affect the accuracy and reliability of the results.

*Operando* investigations of liquid‐based systems are preferred but more challenging. Many advanced characterization techniques, such as X‐ray photoemission spectroscopy (XPS) and scanning electron microscopy (SEM), require high‐vacuum environments, which are incompatible with the organic solvents present in liquid electrolytes. While recent research has explored techniques like in situ XPS with positive pressure to mitigate solvent evaporation, these techniques are, to date, not fully implemented.

#### 1.2.2 The Solid‐State Battery: More Challenging, As All Interfaces are Solid

Solid‐state batteries, by definition, possess unique interfaces as they are all solid (Figure 1b). The electrochemical performance of solid‐state batteries depends on the optimal contact between the electroactive materials, the electronic conductive carbon or alike, and the solid electrolyte, as ion transport and electron transfer occur through these interfaces. However, separating the solid electrolyte (SE) from the electroactive components without damaging the interface is extremely difficult, if not impossible. The SE typically forms a densified layer of solid powder, often sintered using pressure and/or heat, making its removal highly challenging for ex situ investigations. As a result, these interfaces must often be investigated in situ or *in operando*, where the components remain intact.

An alternative approach is to study buried interfaces by accessing the backside of the electrode (current collector side), which is easier to remove than the surface facing the solid electrolyte. This can provide valuable information about the buried interfaces within the composite electrode. However, investigating the surface of the Li metal (or any metallic foil) remains problematic, as the backside of the Li does not directly interact with the chemistry of the solid electrolyte. Therefore, investigating the metal‐SE interface may be difficult. This limitation complicates the study of metal foils, which are critical to understanding the overall electrochemical performance of solid‐state batteries.

A key advantage of solid‐state batteries is their compatibility with vacuum‐based techniques, such as *operando* XPS or SEM, which are highly challenging for liquid‐based systems. However, successful studies require maintaining the integrity of the electrochemical stack during analysis. Minimizing beam‐induced damage is also crucial, as it can alter the observed chemical composition or morphology. Although *operando* techniques offer significant potential for probing these systems, the challenges of ensuring reliable cell contact and preventing beam damage introduce uncertainty into the interpretation of data.

#### 1.2.3 Polymer‐Based Batteries: Dealing with the Adhesive Nature of Polymers

Investigating polymer‐based electrolyte systems (Figure 1c) presents significant challenges due to the adhesive properties of polymer materials, especially at elevated temperatures. At these temperatures, the polymer becomes gel‐like, making it nearly impossible to disassemble without damaging the surface layers and altering their chemistry. This adhesive nature complicates polymer removal and increases the likelihood of surface damage. Furthermore, *operando*‐based techniques, while effective for solids, encounter additional difficulties when applied to polymers. Polymers are inherently sensitive to vacuum environments, often exhibiting outgassing or morphological changes, and are susceptible to beam‐induced damage, which limits their ability to be studied without compromising their integrity. Furthermore, the *operando* measurement requires a regulated temperature setup to ensure proper functioning of the battery polymer electrolyte systems. This adds even further complexity to an already difficult scenario. The interfaces between, for example, current collectors and composite electrodes are often inaccessible without destroying their structure. This architectural complexity makes obtaining reliable and unaltered interfacial data particularly challenging.

These complexities highlight the challenges researchers face in advancing battery technology, particularly in understanding degradation mechanisms. Advanced research and characterization techniques have provided new insights into surface reactions, uncovering previously hidden aspects of battery chemistry. Notably, the chemical composition of most SEI is not homogeneous; recent theories suggest that these gradients arise from multiple physico‐chemical factors.^[^ ^14^, ^15^ ^]^

As illustrated in Figure 1, panels d–f, various analysis techniques enable the investigation of interphases beneath the exposed surface, offering depth profile insights. However, while these methods reveal valuable information about hidden layers, they also come at the cost of altering the surface structure and chemistry.

Mechanical cross‐sectioning, depicted in Figure 1d, involves cutting the sample to reveal depth profiles. This approach is exemplified in Figure 1g, which shows cross‐sectional SEM images of silicon electrodes with a new dual cross‐linked fluorinated binder (FP2SA) binder.^[^ ^16^ ^]^ Figure 1e illustrates the ablation of upper layers through Focused Ion Beam (FIB) techniques, which can be performed at room temperature or under cryogenic conditions to minimize thermal damage. This method allows for highly precise removal of material layer by layer, as demonstrated in Figure 1h, which presents a cryo‐FIB‐SEM cross‐sectional image of a Li/LiPON sample.^[^ ^17^ ^]^ Another approach for accessing the inner layers is the ion milling techniques, shown in Figure 1f, which are often coupled with chemical analysis of the ablated material. Ion milling enables the systematic removal of surface layers to expose underlying structures while performing chemical profiling. An example of this technique is provided in Figure 1i, which shows the FIB‐milled cross‐sectional microstructure of a graphite electrode cycled between 3.00 and 0.02 V at a slow scan rate of 0.05 mV s^−1^.^[^ ^17^ ^]^

All three techniques share the common challenge of altering the sample's chemistry and morphology, requiring careful consideration of these effects during analysis and interpretation.

##### What About the Native Layer?

**Figure 2:**
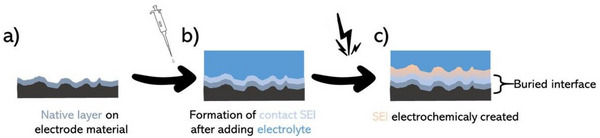
Scheme of electrode surface chemistry in a liquid electrolyte battery, showing the impact of the native surface layer on those upon it formed by chemical and electrochemical means. a) The composite electrode with its surface native layer composed of carbonates, hydroxides, etc.; b) the composite electrode soaked by the electrolyte, with the development of the preliminary SEI here labeled as “contact SEI”; c) composite electrode after being electrochemically cycled, showing the evolution of the native surface layer and the SEI with an indication about the buried interface.

A concern regarding the buried interfaces is the role of the native surface layer that develops once the pristine sample is in contact with air/moisture (prior to cell assembly). Indeed, if an electrode material is in contact with air/moisture (without being classified as air‐sensitive), a thin layer will develop over the exposed surface.^[^ ^13^, ^18^ ^]^ Frequently, carbonate species (e.g., Li_2_CO_3_) or oxide phases are observed at the surface of the sample,^[^ ^19^ ^]^ but then, what will be the fate of this carbonate/oxide formed species along cycling? When considering an electrode material exposed to air before battery assembly, the now formed native layer will be wet by liquid electrolyte during cell assembly. This native layer is likely to transform into a buried interface. More precisely, a so‐called inter*phase*, since the SEI layer resulting from the degradation reaction of the electrolyte will build on top or at the expense of it. It can be deduced that the chemical composition of the native layer, as well as its structure and stability, may vary upon contact with different electrolytes or SEIs, and the way it interacts with its medium remains unclear due to its buried nature (**Figure** **2**). The same logic should apply to solid electrolytes and metal electrodes. For instance, the high reactivity of lithium metal to air and moisture leads to the formation of a dense native passive layer mainly composed of Li_2_CO_3_/LiOH and Li_2_O, as first reported by Aurbach and coworkers^[^ ^20^, ^21^, ^22^ ^]^ and later supported by Shi et al.^[^ ^23^ ^]^ by coupling ellipsometry and ex situ XPS characterization techniques. Some reports address the role of surface native layers on garnet‐type solid electrolyte.^[^ ^24^, ^25^, ^26^ ^]^ With a thickness ranging between one to several tens of nm, depending on the exposure time to air/moisture, this layer has a negative impact on the cycling performance of all‐solid‐state batteries using garnet‐type electrolyte. It was found that the carbonates formed on the surface increase the interfacial resistance leading to internal cell polarization growth. Apparently, this layer and its negative effect can be countered by subjecting the electrolyte to a thermal treatment under inert atmosphere.^[^ ^24^, ^25^, ^26^ ^]^ Another study of the native layer was performed on nano‐based silicon electrode, where the native SiO_2_ layer develops on its surface.^[^ ^27^ ^]^ Interestingly, it was demonstrated that the SiO_2_ layer actively participates in electrochemical processes, leading to several phase transitions, not all of which appear to be reversible. Thus, a legitimate question is whether the native surface layer should be considered the predecessor of a buried interface, or whether it can be considered an electroactive phase participating in electrochemical processes, like the active materials. Figure 2 represents the formation of the SEI on top of the native layer.

##### The Controversial Role of LiF

In papers investigating the SEI or the CEI, LiF is often declared to be one of the main chemical components resulting from the decomposition of certain fluorine‐based salts during cycling. Nowadays, the formation of LiF is extensively debated due to its high sensitivity to beam exposure, especially under XPS and Ar‐ion sputtering. Recently, Gao et al. investigated the parameters that could influence and overexaggerate the role of LiF component in the SEI/CEI.^[^ ^28^ ^]^ Based on their results, one can carefully investigate the surface layer and pay attention to several parameters such as Ar‐ion sputtering time, the lithium carbonate at the surface of the electrode (thus dried storage should be preferred), the measurement time under XPS beam, the vacuum used, the cleaning procedure, etc. These parameters, which are often not reported in the Materials & Methods section of the papers, are crucial for ensuring a proper understanding of surface layer properties and allowing for proper comparison with literature data. Steinrück et al. found that LiF content increases when investigating surface properties of water‐in‐salt electrolyte putting doubt about being a component of the SEI of water‐in‐salt systems.^[^ ^29^ ^]^

Despite the LiF component often being exaggerated in the SEI, it remains a key decomposition product that pushes researchers to develop strategies by developing protective LiF layers. Indeed, bulk LiF possesses unique properties, including high mechanical strength and low solubility. A wide electrochemical stability window (0 to 6.4 V vs Li^+^/Li)^[^ ^30^ ^]^ also contributes to improved long‐term cycling stability of electrode materials. He et al. investigated the formation of ex situ and in situ rich‐SEI, none of them being as efficient as the one generated by the electrolyte decomposition.^[^ ^31^ ^]^ Following this paper, Li et al. discussed the possible approach to improve the SEI properties by adding LiF‐rich layers.^[^ ^32^ ^]^

### 1.3 How Should These Interfaces Be Characterized?

Significant efforts have been put into characterizing the surfaces of battery materials, including composite materials and solid‐state electrolytes. The key to these studies lies in using appropriate characterization tools to reveal the subtle properties of exposed and buried interfaces. In **Table** **1** we summarize the characterization techniques that can be used to probe such interfaces, along with the probe size, what information they can provide, and their main benefits and drawbacks.

**Table 1:** Table summarizing the main characterization techniques used in the literature to probe battery interfaces.

	Technique	Characterization instrument	Resolution expected	Probe and Information	Pros	Cons
Spectroscopic techniques	XPS (X‐ray photoelectron spectroscopy)	Lab‐scale	8–10 nm depth	Exposed surface Chemical composition	Easy to implement Sensitive to surface chemistry	Sample preparation Sensitive to external pollution Ultra‐high vacuum impedes liquid electrolyte use
	HAXPES (hard‐XPS)	Lab‐scale, large‐scale facilities	20 – 50 nm depth	Exposed surface and buried interface Chemical composition	Larger depth analyzed vs XPS	Requires large‐scale facilities Lab scale apparatus are rare
	APXPS (ambient pressure XPS)	Lab‐scale	Sample‐dependent (< 30 nm)	Exposed surface Chemical composition	Similar to XPS Allows use of liquid electrolytes for operando measurements	Not widely implemented Poor signal‐to‐noise ratio Complex experiments
	AP‐HAXPES (ambient pressure HAXPES)	Lab‐scale, large‐scale facilities	Several 10s of nm	Exposed surface and buried interface Chemical composition	Similar to APXPS Allows operando liquid electrolyte measurements	Similar to APXPS Requires large‐scale facilities
	Nano‐XRF (nano‐beam X‐ray fluorescence)	Large‐scale facilities	50–100 nm	Exposed surface Elemental composition	High elemental sensitivity Easy to operate Non‐destructive	Cannot detect very light elements Poor depth profile information Very long measurement times Require large‐scale facilities
	Nano‐FTIR (nanoscale Fourier‐transform infrared spectroscopy)	Lab‐scale	10–20 mm	Exposed surface and buried interface Chemical bonds Atomic coordination and bond length	Identify chemical compounds effectively Lab apparatus and fast collecting time Enables quantitative analysis Non‐destructive	Slow scanning rate for large areas Complex sample preparation
	Micro‐ and nano‐Raman (micro‐/nano‐beam resonance Raman spectroscopy)	Lab‐scale, large‐scale facilities	10 to ≈3 µm	Exposed surface and buried interface Bonding and molecular vibrations	Characterization of amorphous and molecules Local disorder analysis Sensitive to molecular structure Non‐destructive	Complex sample preparation Fluorescence interference Very long measurement time and low peak‐to‐noise ratio Requires large‐scale facilities and specialized equipment for high‐resolution (nano‐Raman)
	ToF (time‐of‐flight)‐SIMS (secondary ion mass spectrometry)	Lab‐scale	<1 to 20 µm	Exposed surface Chemical composition	Extreme surface analysis with very low detection limit Non‐destructive	Requires high vacuum Not suitable for liquid‐based systems
	In situ liquid SIMS	Lab‐scale	20–100 nm	Solid‐liquid interfaces Chemical composition Spatial distribution of species	Similar to Tof‐SIMS *Operando* time‐resolved chemical information at interface	Limited depth profiling through liquid Complex sample preparation, specific cell design Liquid electrolyte susceptible to beam damage
	EDS/EDX (energy‐dispersive X‐Ray spectroscopy)	Lab‐scale (integrated with (S)TEM)	50 to 500 nm (energy‐dependent)	Exposed surface and buried interface Chemical composition	Easily integrated with SEM/TEM for elemental analysis Non‐destructive	Limited resolution and sensitivity for light or too heavy elements
	EELS (Electron Energy Loss Spectroscopy)	Lab‐scale (integrated with (S)TEM)	≈0.1 nm	Exposed surface and buried interface Chemical bonding Electronic structure	High spatial resolution Sensitive to light elements Provides oxidation state and bonding information Non‐destructive	Requires TEM integration Challenging sample preparation Limited to thin samples
	ss‐NMR (solid‐state nuclear‐magnetic resonance)	Lab‐scale	Several mm	Long‐range bulk Molecular structure and dynamics	Detailed atomic/molecular structure information Enables semi‐quantitative analysis Good time resolution Non‐destructive	Poor spatial resolution Extensive signal processing Requires large magnets and dedicated equipment
	Nano‐XAS (nano‐beam X‐ray absorption spectroscopy)	Large‐scale facilities	≈500 nm	Exposed surface and buried interface Chemical composition and oxidation state Local atomic environment	Surface dependence Chemical and electronic structure Non‐destructive	Low signal‐to‐noise ratio Requires access to large‐scale facilities
Imaging techniques	(cryo)‐SEM (scanning electron microscopy)	Lab‐scale	50 to 500 nm (energy‐dependent)	Exposed surface and buried interface Particle size and morphology	Attenuation of beam effect while using cryo stage Chemical contrast Non‐destructive	High vacuum required Not suitable for liquid samples
	(cryo)‐TEM (transmission electron microscopy)	Lab‐scale	1 to 100 nm (energy‐dependent)	Exposed surface and buried interface Crystalline structure	Preserves native layers High‐resolution options for single‐crystal structure refinement Non‐destructive	Averaged information across probe volume Requires high vacuum Not suitable for liquid samples
	(cryo)‐FIB (focused ion beam)	Lab‐scale	10 nm to 10 µm	Surface, buried interface and bulk Morphology and topography	Enables profiling of sensitive samples Complementary to SEM/TEM	Low signal‐to‐noise ratio Not suitable for liquid/gas samples Destructive technique
	AFM (Atomic Force Microscopy)	Lab‐scale	≈1 – 10 nm	Exposed surface Topography Mechanical and electrochemical properties	High spatial resolution Can operate in various environments (gas, liquid, vacuum) Non‐destructive	Limited to surface analysis Slow scanning rate for large areas Complex sample preparation
Diffraction techniques	Surface (grazing angle) and micro XRD (X‐ray diffraction)	Lab‐scale, large‐scale facilities	A few nm to 50 µm	Surface, buried interface, and bulk Crystalline structure	Defect, strain, and microstructure analysis Non‐destructive	High uncertainty for light elements Unreliable for amorphous materials
	XRR (X‐ray reflectometry)	Lab‐scale, Large‐scale facilities	≈0.5–500 nm	Exposed surface and buried interface Crystalline structure	Sub‐nanometer spatial resolution Defect, strain, and microstructure analysis Non‐destructive	High uncertainty for light elements Unreliable for amorphous materials
	NR (neutron reflectometry)	Large‐scale facilities	≈0.5–500 nm	Exposed surface and buried interface Crystalline structure	Complementary to XRR Sub‐nanometer spatial resolution Mixed occupancy and vacancy analysis Non‐destructive	Low resolution compared to synchrotron XRR Requires access to large‐scale facilities Isotope substitution often required Unreliable for amorphous materials Restricted for certain radiative elements

A particular focus: photoelectron spectroscopy techniques

**Figure 3:**
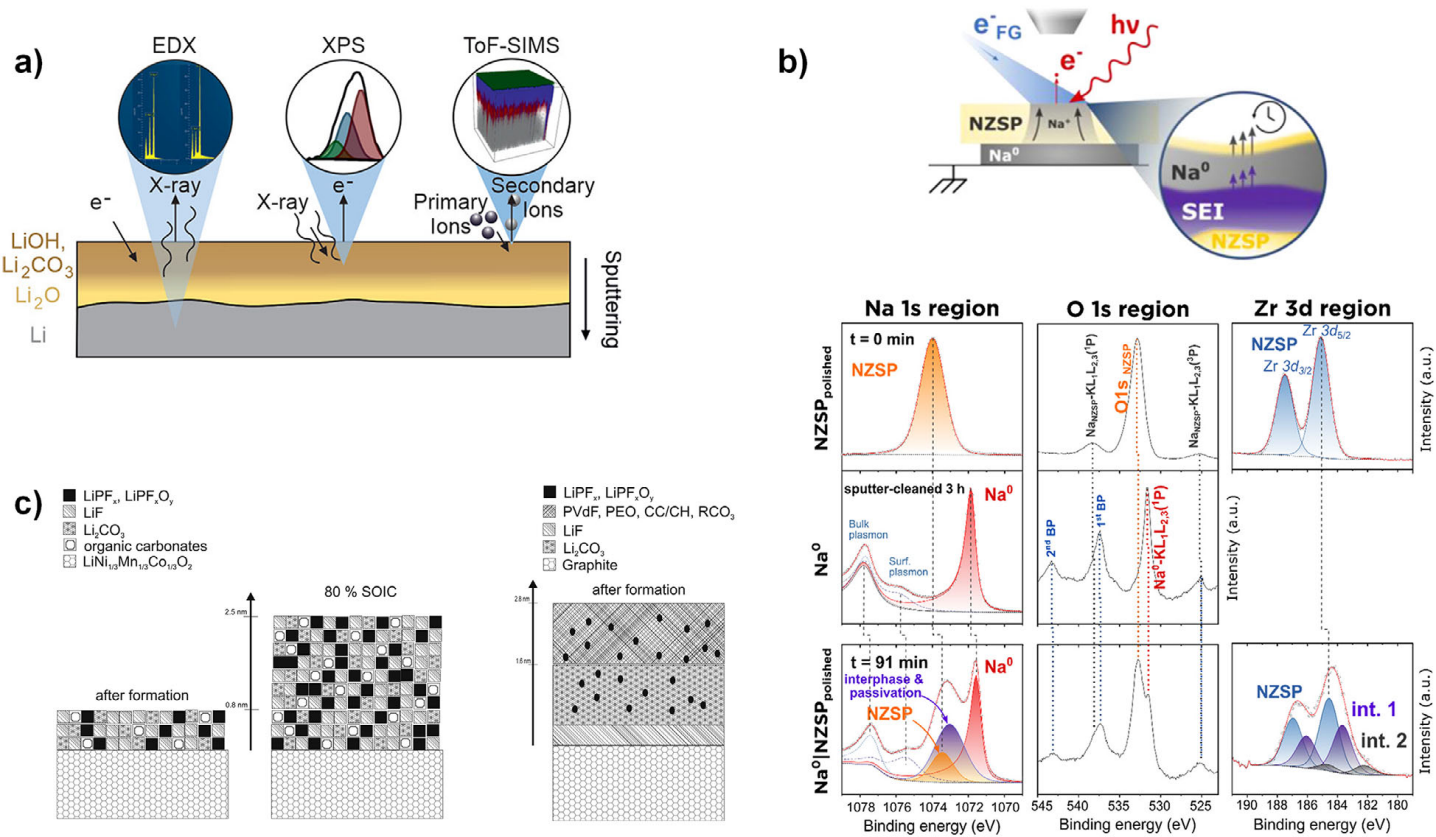
Examples representing the results obtained from several surface characterization techniques. a) Graphical representation of the three main techniques used for thin surfaces and passivation layers characterization. The scheme summarizes the principle of measurement and the depths that can be reached with Energy dispersive X‐ray spectroscopy (EDX), X‐ray photoelectron spectroscopy (XPS), and time‐of‐flight secondary‐ion mass spectrometry (ToF‐SIMS). Reprinted from ref. [43] Copyright (2021) American Chemical Society: b) XPS Results obtained after surface characterization of Na | NZSP interfaces. A schematic representation of the XPS analysis configurations is shown. A charge‐compensating flood gun (FG) of the instrument was used and employed to supply electrons for the sodium plating reaction on the NZSP surface and type of XPS data collected during the experiment and fitting model used for interpretation (not at scale). Adapted from ref. ^[^ ^44^ ^]^; c) Proposed model for passivation layers formed on the surface of grains of NMC111 and graphite for both positive and negative electrode after formation. The local chemistry and layers thickness have been obtained by XPS with sputter depth profiling investigation. Reprinted from ref. [45] Copyright (2013) American Chemical Society.

To date, XPS remains the technique of choice for probing the chemical nature of the surface layer due to its considerable depth profile resolution of 8–10 nm^[^ ^33^ ^]^ (**Figure** **3**a). Ex situ and *post mortem* XPS have been used to describe the chemical nature of the SEI.^[^ ^34^, ^35^ ^]^ An important drawback when using XPS in battery research is the need of an ultrahigh vacuum (UHV) chamber,^[^ ^36^ ^]^ which makes tracking the dynamic formation of the SEI in liquid‐based electrolytes nearly impossible.

Recent advancements toward near‐ambient‐pressure XPS have partially lifted this constraint. However, this technique is relatively rare and not widely available in laboratories. Furthermore, two additional challenges must be considered: 1) the cell design, which must cycle under very low pressure within the XPS chamber, and an open lid is required to allow the beam to scan the sample, and 2) the local charging effect caused by cycling, which influences the peak position and assignment.^[^ ^37^, ^38^, ^39^ ^]^

XPS investigations have been carried out with *operando*‐like measurements to follow the degradation processes that occur between LiCoO_2_ and Li_3_PS_4_ solid electrolyte upon oxidation.^[^ ^40^ ^]^ The same authors performed similar measurements comparing ex situ data samples and *operando* measurements on a Li_4_Ti_5_O_12_ electrode in contact with a Li_3_PS_4_ solid electrolyte.^[^ ^41^ ^]^ An important conclusion the authors reach is that *operando* measurements are more reliable than ex situ ones for understanding surface stability. This is because ex situ measurements require cell disassembly, which may lead to surface modification.

Secondary Ion Mass Spectrometry (SIMS) offers powerful capabilities for probing battery interfaces. For instance, Zhou et al.^[^ ^42^ ^]^ utilized in situ liquid‐SIMS to observe the real‐time chemical evolution of the SEI directly in a liquid electrolyte, identifying dynamic component changes despite challenges like the complex vacuum‐liquid interface. Complementing such *operando* approaches, ex situ time‐of‐flight SIMS (ToF‐SIMS), as used by Otto et al.,^[^ ^43^ ^]^ provides in‐depth chemical characterization of passivation layers formed on lithium‐metal surfaces, offering very low detection limits for detailed surface analysis under high vacuum conditions, though careful sample transfer is crucial to preserve interface integrity.

While XPS remains the technique of choice for surface investigation, its limited probe thickness (typically 8–10 nm) can be insufficient for fully characterizing surface layers, especially when it is referred to buried interfaces. The depth‐profiling approach is required to “dig” under the SEI to reveal the hidden interface.^[^ ^39^ ^]^ Such an approach requires i) tuning the incident X‐ray beam energy and incidence angle at the laboratory scale (Figure 3b) or using synchrotron sources (hard‐XPS or HAXPES) and ii) tuning the sample preparation by either mechanical fracturing or ion etching. As an example of the former, Philippe et al.,^[^ ^46^ ^]^ by increasing the energy of the incident beam using XPS and HAXPES, highlighted the evolution of the chemistry and thickness of the passivation layer on an Si electrode during cycling. As an example of the latter approach to dig into the sample, Niehoff et al.^[^ ^45^ ^]^ found differences in the SEI composition as a function of depth profiling, as shown in Figure 3c. However, they found that sample preparation was likely to alter the sample chemistry owing to Ar etching (damage caused by the ion beam). This technique generally vaporizes the chemical species near the surface, which later redeposit on the surface to be further investigated, altering the results.^[^ ^47^ ^]^ Unfortunately, XPS is limited in its spatial resolution and the information that can be gathered since only the chemical nature of the surface layer can be probed.

X‐ray and neutron reflectometry are surface‐sensitive techniques that can probe the depth profile of an electrode within the nanometer range and provide information about reactions process.^[^ ^13^ ^]^ The main issue with this technique is the surface roughness of the sample, which should be minimized or avoided because the signal quality might degrade.^[^ ^48^ ^]^ In the past, this issue was addressed using either thin‐film electrodes sputtered on a current collector^[^ ^49^, ^50^ ^]^ or by probing single‐crystal samples,^[[^ ^51^ ^]^ both of which are far from practical battery application. Interestingly, reflectometry techniques coupled with spectroscopy techniques have been used to reveal an underlying layer of the SEI attributed to a buried interface, but its visualization and intrinsic existence are a matter of debate in the literature due to the difficulties in reproducing the results.^[^ ^50^, ^52^ ^]^

**Figure 4:**
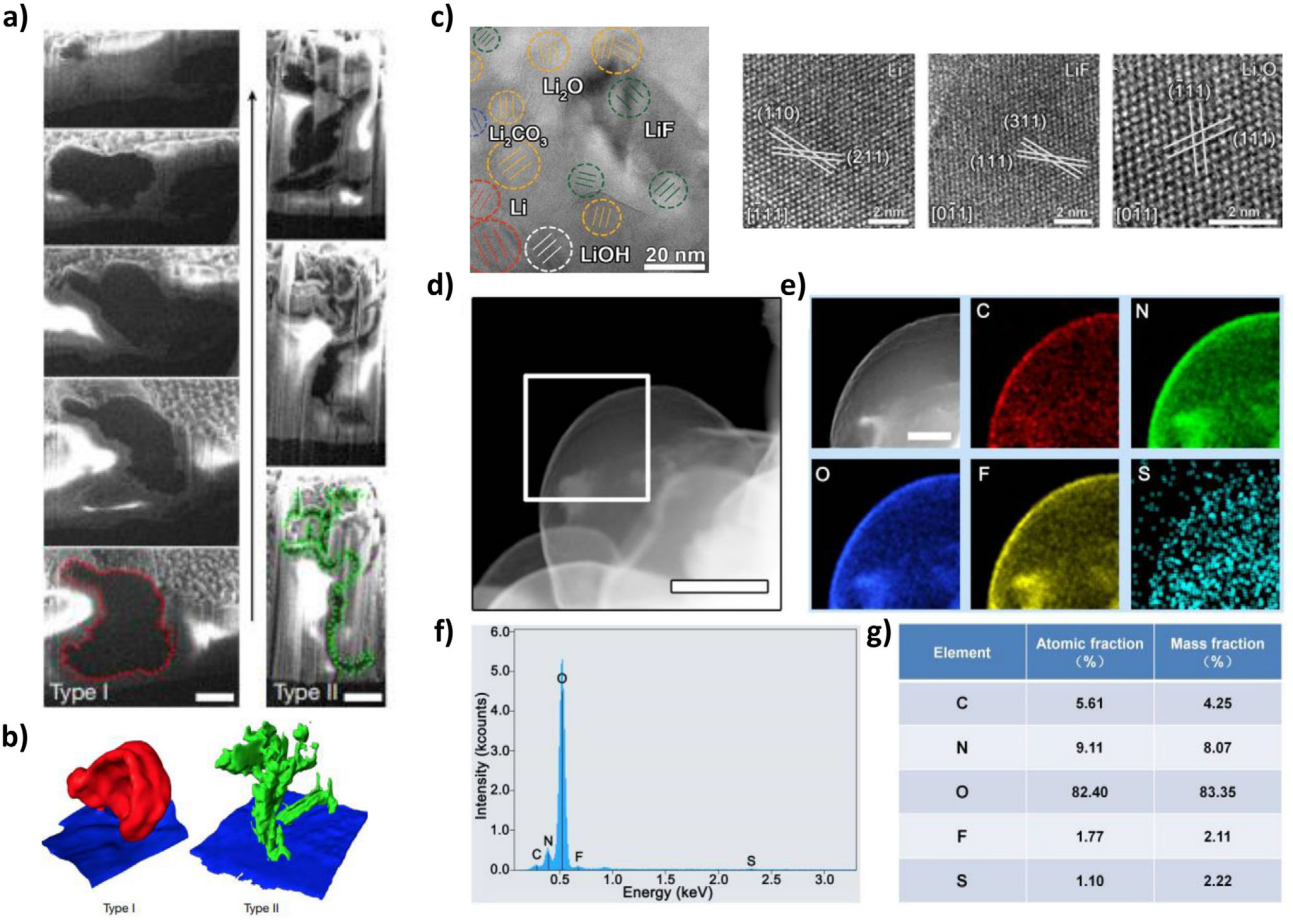
Characterization of electrode materials using cryo‐stage. a) Cross‐sectional images produced by cryo‐FIB and cryo‐scanning electron microscopy (cryo‐SEM) showing two distinct dendrite morphologies. Reprinted from ref. [55] b) 3D reconstructions of the observed dendrite shown in panel (a). Reprinted from ref. [55] c) Cryo‐TEM characterization of Li‐PEO interface, with the corresponding HRTEM images of Li, LiF, and Li_2_O (from left to right) showing long‐range ordering lattice. Reprinted from ref. [56]. Copyright John Wiley and Sons. d) Characterization of SEI formed at −0.05 V without TESM. Cryo‐STEM image of the Li deposited at −0.05 V without TESM in ether‐based electrolytes at 1 mA cm^−2^ with a capacity of 0.5 mAh cm^−2^. e) The corresponding elemental mapping images of the area highlighted in (d). Scale bars, (d) 500 nm and (e) 200 nm. f) The spectrum of element intensity obtained from (e). g) The relevant atomic and mass fraction of varied elements measured in (e). Panel (d tog) are reprinted from ref. [57]

Nanometer‐thick layers can be tracked using high‐resolution imaging techniques such as transmission electron microscopy (TEM). Given the chemical sensitivity of the interfacial layer and its ability to suffer beam damage, cryogenic electron microscopy (cryo‐EM) is generally preferred owing to its high nanometer‐scale resolution and cryo‐stage that normally preserves the surface layer, as shown by Li et al.^[^ ^53^ ^]^ Thus, the growth mechanism of the Li dendrites can be reconstructed (**Figure** **4**a,b). Alternative cryo‐imaging techniques are often employed to investigate metallic foils. Cryo‐techniques such as FIB‐SEM imaging coupled with energy dispersive X‐ray spectroscopy (ESD) analysis or electron energy loss spectroscopy (EELS) analysis (Figure 3d–g) allow the monitoring of the cross‐sections of metallic lithium cycled in different electrolytes;^[^ ^54^ ^]^ however, the main challenge of cryo‐TEM remains the complex sample preparation, which can be easily altered by diverse pollutants. To date, there are no commercialized cryogenic and airtight transfer chambers, and the number of publications in this area remains low, even though they allow the direct observation of cross‐sections and thereby the interphase chemistry of all battery systems (Figure 4c).

As shown, a tremendous number of advanced techniques are capable of probing interfaces, some of which are perfectly established in the field of batteries and others are starting to be intensively used to effectively provide missing information on interfacial processes. The next section shows the outcome of the investigation performed on both types of interfaces: the one generated from the organic electrolyte and that formed by the inorganic electrolyte.

### 1.4 Beam Damage and Related Artifacts

Many surface components are highly sensitive materials due to their reactive chemical composition and minimal thickness. However, these are not the only samples exposed to unwanted alteration. Several papers are reporting not only surface but also bulk damage during the investigations performed in situ, *operando* and ex situ. By investigating the bulk of an electrode material, Black et al. observed beam damage on an NMC electrode, which delayed the electrochemical performance of the cell when the sample was exposed to the beam. Similar conclusions were obtained by Christensen et al., who proposed strategies to overcome beam damage.^[^ ^58^, ^59^ ^]^ Jäckel et al. proposed some approaches to overcome the beam damage, as well. During TEM‐EELS investigation, the effects caused by the electron beam can be reduced by tuning the exposure time and voltage/current parameters. Fantin et al. investigated the beam damage induced by XPS analysis occurring at lab scale and at large scale facilities.^[^ ^60^ ^]^ At the lab scale, the impact of beam damage can be reduced by reducing the measurement time whereas at the synchrotron, it is better to take snapshot of the sample to reduce the beam damage impact. Recently Koh et al. performed an investigation of the SEI properties using cryogenic electron microscopy and observed that SEI are largely amorphous and that they are extremely beam‐sensitive and thus prone to beam‐induced artifacts.^[^ ^61^ ^]^ By using nanobeam diffraction, they observed an unaltered structure of the SEI.

Thus, one must be cautious when discussing SEI properties, their chemistry, and physico‐chemical properties. As demonstrated, the surface layers are sensitive to the working environment as well as the way they are handled and further characterized. Prior investigation should be systematically carried out on a sample to assess the impact of beam damage before drawing conclusions. Similarly, the sample preparation should be properly explained in the literature data as it can influence the chemistry of the surface layer. Both beam damage and inadequate sample preparation represent current limitations in literature, hindering proper comparison of results due to missing information.

### 1.5 Organic Versus Inorganic Interfaces

Despite extensive research and numerous studies on interfaces, gaps in our understanding of interfacial properties persist. To date, the optimal combination for prolonged cycling of a positive electrode material, negative electrode material, and electrolyte bridging remains elusive. Multiple factors hinder progress. First, it is difficult to compare reports, even when using identical materials, due to parameters beyond our control. Second, accessing the interface layer is problematic and risks degradation during investigation. Finally, native surface layers on active materials, solid electrolytes, current collectors, binders, and conductive agents significantly impact the nature of the interface layer.

To address the differences, the scientific community has established a common ground with respect to interface layers, particularly evident when comparing organic and inorganic electrolytes, as illustrated below.

#### 1.5.1 Interface Properties with an Organic Electrolyte

**Figure 5:**
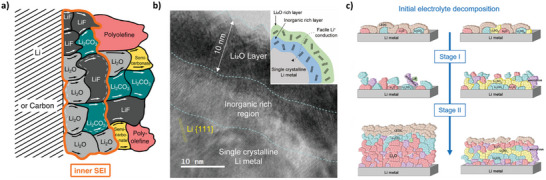
Different SEI representations depicted in the literature. a) The polyhetero microphase as was conceived by Peled et al.^[^ ^66^ ^]^ Reproduced with permission of IOP Publishing Ltd. Copyright (1997) The Electrochemical Society. The panel a) was revisited recently by Krauss et al.,^[^ ^67^ ^]^ under CC BY‐NC‐ND 4.0 licensing; b) Cryo‐HRTEM image of the dual‐layered inorganic‐rich SEI formed in the high‐concentration DME electrolyte, and a schematic of the structure of the dual‐layered inorganic‐rich SEI (inset). Figure by Wi et al.,^[^ ^64^ ^]^ reproduced under CC BY‐NC‐ND 4.0 licensing; c) left side column, SEI evolution without additives in the ethylene diethyl carbonate electrolyte, with accumulation of lithium oxide (Li_2_O) coming from the continuous decomposition of lithium ethylene dicarbonate (LEDC) and lithium carbonate (Li_2_CO_3_) formed on the surface on the Li metal; right side column: the effect of ethylene sulfate as electrolyte additive, namely the deposition of lithium‐sulfur species, such as Li_2_SO_4_, that form a layer (represented in yellow) that prevents further decomposition of Li_2_CO_3_. Reprinted from ref. [68] Copyright (1999–2024) John Wiley & Sons.

In general, the community agrees that the SEI layer is a result of the decomposition of an organic electrolyte on the surface of electrode materials. This layer consists of an inner layer made of inorganic products and an outer layer made of organic products,^[^ ^62^, ^63^ ^]^ as shown in **Figure** **5**a. Depending on the solvent employed in the electrolyte, the outer layer could be mostly composed of inorganic compounds, particularly in the case of reduction‐resistant solvents such as dimethoxymethane or dimethoxyethane (DME)^[^ ^64^ ^]^ (Figure 5b). The inner inorganic layer is usually described as an intricate mosaic of decomposition products mainly originating from the electrode side that gradually transition into the bulk material of the electrode. The thickness of the SEI layer can also be tuned by employing well‐known electrolyte additives, such as fluoroethylene carbonate (FEC) and vinylene carbonate (VC) as presented in Figure 5c. Recent strategies in electrolyte engineering, such as the development of synthetic sulfonamide‐based solvents with electrochemically active additives, aim to create more robust SEI layers capable of withstanding high voltages in lithium‐metal batteries, thereby improving electrochemical performance, safety, and longevity.^[^ ^65^ ^]^ In any case, the SEI has to be lithium ion‐conducting to allow lithium‐ion transport through the layer and into the negative electrode, but also electronically insulating.

The physicochemical properties of the SEI remain complex, and the SEI formed from organic liquid electrolytes is governed by many parameters such as the nature of the solvent, salt concentration, and possible—but not mandatory—electrolyte additives. Additional parameters can also play a role, such as electrode engineering (binder content and conductive agent), operation and storage temperatures, and electrochemical potential at which SEI is formed.^[^ ^69^ ^]^ Several reviews^[^ ^15^, ^70^ ^]^ have described in detail the SEI properties, and some have demonstrated the impact of advanced characterization techniques to improve SEI understanding.^[^ ^71^, ^72^, ^73^ ^]^ To date, the SEI has been thoroughly investigated, particularly with respect to i) novel challenging electrodes such as silicon electrodes and Li metal, and ii) the evolution of the SEI upon aging. The former aims to understand how the dynamics of the SEI are affected by the large volume changes experienced by the electroactive materials.^[^ ^74^, ^75^ ^]^ For the latter, the target of long‐term cycling is to shed light on the fate of the SEI upon cycling,^[^ ^76^, ^77^ ^]^ particularly regarding cross‐talk contamination of the SEI.^[^ ^78^, ^79^ ^]^

The SEI is a concept used exclusively to describe the interfacial reactions occurring at the surface of negative electrode materials. For positive electrode materials, several concepts and terminologies have been used, such as the solid polymer interface (SPI); however, the CEI is dominant in the literature.^[^ ^80^, ^81^ ^]^ In basic principle, the concept of the CEI is similar to that of the SEI: the organic electrolyte is oxidized and decomposed at the surface of the positive electrode material, so at the first approximation, similar investigations with similar techniques have been carried out.^[^ ^78^ ^]^ However, key differences exist between the SEI and the CEI, primarily that the CEI can dissolve during cycling, leading to the well‐known “cross‐talk” contamination of the negative electrode and exposing a “fresh” surface on the positive electrode material to new CEI deposition.^[^ ^82^ ^]^ This process results in lithium consumption, which is particularly detrimental in full cells where lithium is limited.^[^ ^83^ ^]^ As the CEI is strongly dependent on the operating voltage, at potentials beyond the range of 4.2–4.3 V versus Li^+^/Li using an organic‐liquid‐based electrolyte, the CEI will be even more pronounced. Therefore, it is of utmost importance to optimize the electrolyte formulation, the electrolyte additive, and the surface protection of the electroactive material.^[^ ^84^, ^85^ ^]^

While the CEI is generally the main interfacial layer discussed when it comes to positive electrode materials, it is important to recognize that the concept of *buried interfaces* at the positive electrode extends beyond the CEI surface layer alone. The complexity of these subsurface regions makes broad generalizations difficult. Interfaces between the active positive electrode material and other composite components—such as conductive additives and binders—also fall within this buried interface definition, particularly as they may become progressively enveloped or chemically altered due to electrolyte infiltration and CEI growth during cycling. Moreover, the native surface of the active material may respond differently to interphase formation than the bulk, introducing further heterogeneity. Structural rearrangements, phase transformations, or the formation of resistive layers at the immediate surface of the positive electroactive material—sometimes underlying or intermixed with the CEI—add additional complexity to this interfacial region. These aspects are often challenging to distinguish from the CEI itself but play a crucial role in dictating charge transfer kinetics, ionic diffusion pathways, and ultimately, the long‐term electrochemical performance and degradation of the positive electrode. Model‐based electrodes are then of utmost importance if one wants to understand better the possible intricated interfaces.

Interfaces relying on organic electrolytes have been intensively investigated as they were the first ones being implemented in Li‐ion battery technology.^[^ ^1^ ^]^ Over time, due to the limitations they presented in terms of stability and safety, alternative electrolytes have been developed, mostly relying on inorganic electrolytes.

#### 1.5.2 Interfacial Properties with an Inorganic Electrolyte

Among the alternatives to organic liquid electrolytes, solid electrolytes based on oxides, mostly ceramic‐type (Li_7_La_3_Zr_2_O_12_ (LLZO), Li_7_La_3_Ti_2_O_12_ (LLTO), Li_1.3_Al_0.3_Ti_1.7_(PO_3_)_4_ (LATP), lithium phosphorus oxynitride (LiPON), etc.), thiophosphate materials (Li_3_PS_4_ (LPS), Li_6_PS_5_Cl (LPSCl), Li_10_GeP_2_S_12_ (LGPS), etc.), and polymer‐type (polyethylene oxide (PEO)‐LiTFSI, etc) have been developed to tackle safety issue. Similarly, aqueous electrolytes are possible alternatives for overcoming the safety limitations of organic liquid electrolytes. Water‐in‐salt electrolytes have emerged as an alternative and safer solution because the electrochemical stability window of water can be extended using salt saturation. Although the precise nature of the interfacial reaction is still under debate, involving either LiF^[^ ^86^, ^87^ ^]^ or LiOH^[^ ^88^, ^89^ ^]^ derivatives, there seems to be a consensus on the complexity of the interphases formed in water‐in‐salt systems, as summarised in the review by Jommongkol et al.^[^ ^90^ ^]^

Beyond the primary physical 2D interface (electrode‐electrolyte junction), the internal 3D architecture of composite positive electrodes in inorganic electrolyte systems presents a complex network of buried interfaces that are critical to electrochemical performance. Recent research efforts have increasingly focused on elucidating the phenomena at these active material/electrolyte, active material/conductive additive, and electrolyte/conductive additive interfaces.

For instance, the electroactive material/SE interface is susceptible to both electrochemical and chemo‐mechanical degradation. Side reactions between common oxide layered electrode (e.g., NMC, Li_2_CO_3_) and sulfide solid electrolytes typically lead to the formation of resistive interlayers, increasing.^[^ ^91^, ^92^, ^93^ ^]^ Moreover, the volume changes experienced by the electroactive material particles during cycling can induce stress, leading to delamination from the SE, formation of voids, and loss of ionic pathways.^[^ ^94^ ^]^ Advanced techniques such as operando X‐ray diffraction (XRD), X‐ray tomography (XRT), FIB‐SEM, and high‐resolution TEM are being employed to visualize these dynamic changes and understand contact loss mechanisms.^[^ ^17^, ^95^, ^96^, ^97^, ^98^, ^99^ ^]^

Except for some solid‐state technologies, such as LiPON‐based batteries, which are currently commercialized, many others are still at the R&D level because of the numerous unknown phenomena occurring at the interfaces. Investigations on the Li‐LiPON interface have yielded different results depending on the techniques used, again highlighting that these studies are difficult to compare. Using XPS, (S)TEM, and focused ion beam/scanning electron microscopy (FIB/SEM) coupled with EDS mapping, Cheng et al.^[^ ^98^ ^]^ observed a thick interphase of ≈ 80 nm composed of N‐rich lithium compounds. Hood et al.^[^ ^99^ ^]^ found an interphase with a similar thickness, but identified a compositional gradient in their in situ experiments by employing TEM and EELS. The transition revealed that the P‐rich compounds near the LIPON surface evolved into a phosphorous‐free, O‐rich layer that seamlessly integrated with the Li surface. In contrast, Browning et al.^[^ ^100^ ^]^ agreed with other researchers on composition; however, while performing in situ neutron reflectometry (NR) experiments, they discovered a very thin interphase layer (≈ 7 nm) between Li and LiPON. Despite these three parallel investigations, differences in chemical composition, thickness, and other properties remain, highlighting the importance of better standardization protocols and sample preparation.

Only a few research groups have investigated the interfacial properties of polymer‐like technology; Xu et al. investigated SEI formation at the graphite/PEO‐LiTFSI interface.^[^ ^101^ ^]^ They conducted an ex situ XPS investigation on both sides of the interface, which revealed changes in the interfacial compositions. This investigation indicated a gradient composition with higher salt degradation on the graphite electrode surface. He et al.^[^ ^10^ ^]^ employed multiscale in situ microscopy and Fourier‐transform infrared (FTIR) spectroscopy to scrutinize the graphene/PEO‐LiTFSI interface. Their attenuated total reflection (ATR) and near‐field FTIR observations, conducted before, during, and after Li plating/stripping, revealed that non‐uniform Li deposition influenced SEI formation. Notably, they observed a localized polymer phase transformation at the electrode surface, shifting from a crystalline state to an amorphous one, possibly due to localized polymer chain decomposition or specific interactions between the graphene/SEI and the polymer.

**Figure 6:**
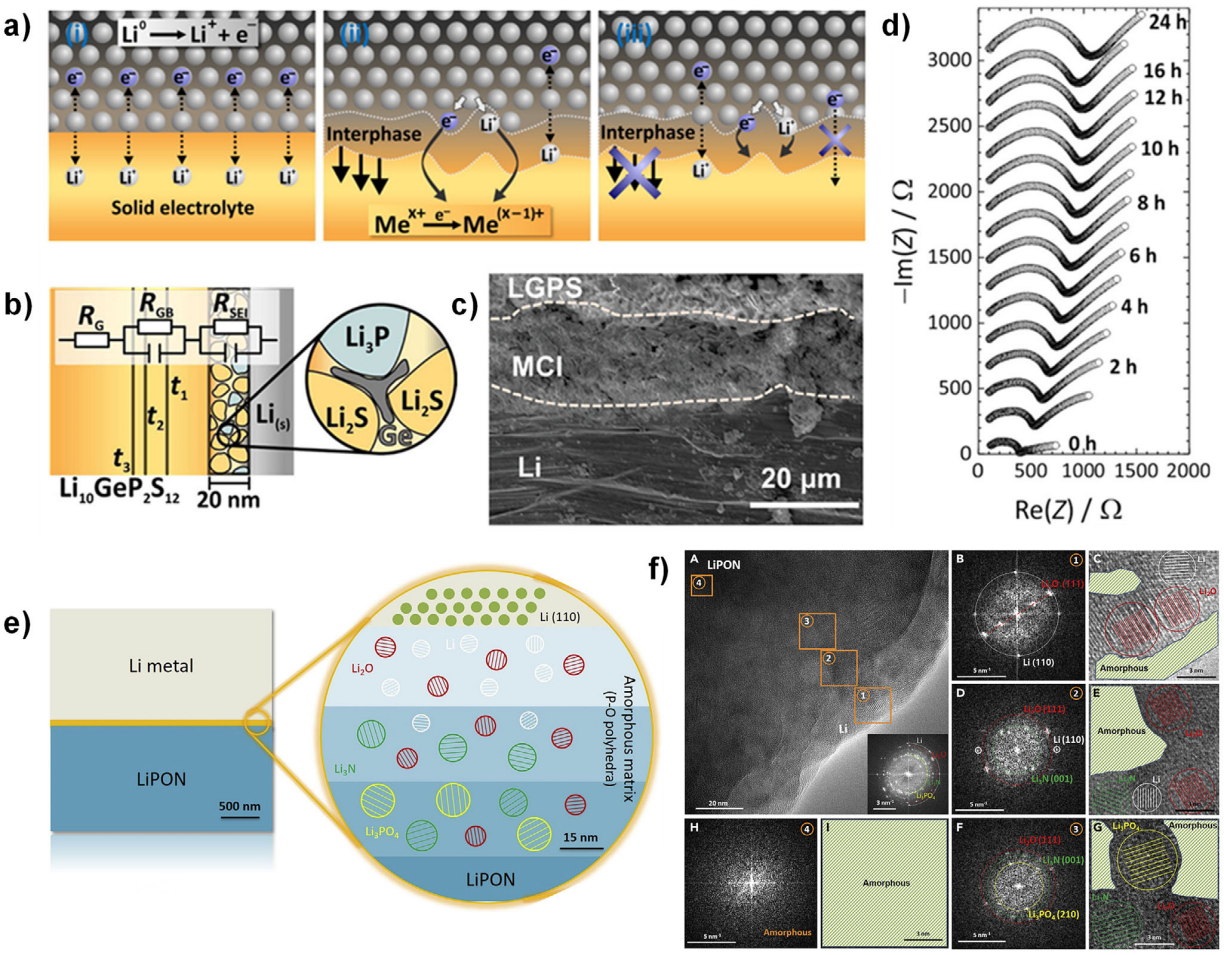
Scheme representing the interfaces in solid‐state batteries. a) Interfaces found between lithium metal and a solid superionic conductor of Li^+^. i) Thermodynamically stable interface where no reaction occurs; ii) electronic‐ionic mixed conducting interphase (MCI) that enables decomposition; iii) metastable SEI, kinetically stabilized. Reprinted from ref. ^[^ ^92^ ^]^, with permission from Elsevier. b) Schematic of the interphase formation at the Li/Li_10_GeP_2_S_12_ interface. The decomposition leads to different binary compounds with poor transport properties, such as Li_3_P, Li_2_S, and Ge (or Li_15_Ge_4_), leading to an increasing interfacial impedance with time. c) Cross‐sectional SEM images of Li|LGPS|Li symmetric cell after resting for 40 h. Reprinted from ref. ^[^ ^105^ ^]^, Copyright (1999–2024) John Wiley & Sons. Panels (b,d) reprinted with permission from ref. ^[^ ^93^ ^]^, Copyright (2016) American Chemical Society. e) Representation of the interfacial amorphous matrix between Li metal and LiPON upon contact. The electrochemical potential of lithium‐ion ${\tilde{\mu }}_{Li^{+}}$, the electrochemical potential of electron ${\tilde{\mu }}_{e^{-}}$, and the chemical potential of lithium *µ*_*Li*_, are presented. f) (A) HRTEM image of the interphase in LiPON batteries where four regions (regions 1–4) are highlighted by orange squares to indicate different stages of the multilayered structure across the interphase. Inset image is the FFT result of the whole area in (A). (B,D,F,H) FFT patterns corresponding to regions 1–4 as highlighted in (A), respectively. (C, E, G, and I) Nanostructure schematic overlaying the HRTEM images that correspond to regions 1–4 as highlighted in (A), respectively. e,f) reprinted from ref. ^[^ ^98^ ^]^, with permission from Elsevier.

For inorganic‐based electrolytes, interfacial investigations are even more challenging because the dismounting of cells is far from trivial and is most likely to bias the results owing to a lack of reproducibility in the cell handling. Some solid‐state materials such as thiophosphate‐based materials pose additional challenges owing to their air sensitivity and beam damage susceptibility. However, their interfaces have been thoroughly investigated, as mentioned in this review,^[^ ^102^ ^]^ and referred to the mixed‐conducting interface (MCI,^[^ ^92^, ^93^ ^]^ **Figure** **6**a). Illustrative examples of such MCI interfaces are presented in Figure 6b–d for Li_10_GeP_2_S_12_ (LGPS) solid electrolyte. In contrast, the LiPON electrolyte exhibits a different scenario, forming a kinetically stable interfacial amorphous matrix with Li metal, as depicted in Figure 6e,f.

Argyrodite materials are prone to decomposition due to beam damage, which can result in biased results during investigation, particularly when using focused, high‐energy sources like TEM. Alternatives, such as ToF‐SIMS, enable indirect analysis.^[^ ^103^ ^]^ Hence, many techniques must be coupled to provide complementary insights into the chemistry of the interfaces. A successful example of this is a report published by Riegger et al., who studied the thiophosphate Li_7_SiPS_8_ by coupling in situ ToF‐SIMS measurements with solid‐state NMR spectroscopy.^[^ ^104^ ^]^ They found that the formation of Li_3_P phases and Li–Si alloys conferred the properties of a mixed ionic and electronic conductor to the interface.

The role of conductive additives, typically carbon, introduces further interfacial complexity. While essential for electronic percolation, direct contact between carbon and certain SEs, like sulfides, can trigger electrochemical decomposition of the SE, especially at higher voltages, forming insulating byproducts.^[^ ^106^, ^107^ ^]^ Recent studies investigate strategies to mitigate this, such as tuning the carbon content and distribution, exploring alternative conductive agents, or designing novel electrode architectures to minimize detrimental SE‐carbon reactions while maintaining electronic conductivity.^[^ ^106^ ^]^ Techniques like scanning spreading resistance microscopy (SSRM) are emerging to map local electronic and ionic conductivity distributions within these composite structures, providing insights into the functionality of these buried contacts.^[^ ^108^ ^]^

Elucidating these intertwined chemical, electrochemical, and mechanical phenomena at the various interfaces, buried or not, within the composite electrode is a key focus. This often requires a combination of advanced ex situ, in situ, and *operando* characterization techniques, coupled with computational modeling, to build a comprehensive understanding.^[^ ^109^ ^]^

The preceding discussions highlights the diverse nature of interfaces and interphases across various battery chemistries. The inherent complexities in their structure, resultant properties, physical accessibility, and the necessary characterization strategies are markedly different depending on whether a liquid, solid‐state, or polymer‐based electrolyte system is employed. **Table** **2** summarizes these critical distinctions. It compares the typical SEI and CEI structures, key interfacial properties including accessibility for analysis, and the tailored characterization approaches demanded by each system.

**Table 2:** Comparison of interphases (SEI/CEI) and interfacial characteristics in different battery systems.

Feature	Liquid Electrolyte System	Solid Electrolyte System	Polymer‐based Electrolyte System
Interphase Structure (SEI/CEI)	**SEI (Negative Electrode)**: Forms from the electrolyte reduction. Typically, a multi‐layer structure with an inner inorganic layer (e.g., Li₂CO₃, LiF, Li₂O) and an outer organic layer (e.g., polymers, oligomers, lithium alkyl carbonates). The outer layer can be inorganic‐rich with certain solvents (e.g., DME). Thickness can be tuned by additives. **CEI (Positive Electrode)**: Forms from electrolyte oxidation; can be similar in concept to SEI but may dissolve during cycling, leading to cross‐talk. Composition depends on electrolyte and voltage.	**SEI/CEI & Other Interfaces**: Interphases form at electrode/solid electrolyte physical interface (2D). For example, at Li/LiPON interfaces, an ≈80 nm interphase rich in N‐compounds or a P‐rich to O‐rich gradient has been observed, while a thinner ≈7 nm layer was also reported. Sulfide SEs can react with oxides active materials forming resistive interlayers. Thiophosphates like Li₁₀GeP₂S₁₂ (LGPS) can form a mixed‐conducting interphase (MCI) with Li metal, decomposing into Li₃P, Li₂S, Ge (or Li₁₅Ge₄).	**SEI/CEI & Other Interfaces**: SEI forms at the negative electrode with intervention of the polymer electrolyte., E.g., graphite/PEO‐LiTFSI react by forming a compositional gradient, showing higher salt degradation on the graphite side. Localized polymer phase transformation (crystalline to amorphous) at the electrode surface are also observed during Li plating/stripping, possibly due to polymer chain decomposition or interactions with graphene/SEI.
Interfacial Properties & Accessibility	**Accessibility**: Interfaces are generally easy to access for ex situ analysis after cell disassembly due to clear liquid/solid phase separation and removable separator. **SEI/CEI Properties**: SEI must be Li‐ion conducting and electronically insulating. CEI dissolution can lead to Li consumption. Native layers on electrodes transform into buried interphases upon electrolyte contact.	**Accessibility**: Extremely difficult to separate SE from electroactive components without damaging the interface. Accessing the backside of the electrode (current collector side) is an alternative. Interfaces are often buried. **Interface Properties**: Performance depends on optimal contact between active materials, conductive carbon, and SE. Interfacial resistance can increase due to surface carbonates on garnet electrolytes or resistive interlayers with sulfide SEs. MCI formation can occur.	**Accessibility**: Very challenging due to the adhesive nature of polymers, especially at elevated temperatures where they become gel‐like, making disassembly without surface damage nearly impossible. Interfaces between current collectors and composite electrodes are often inaccessible without destroying their structure. **Interface Properties**: Adhesive nature complicates removal and increases likelihood of surface damage.
Characterization Approach	‐ **Ex situ**: Common due to ease of disassembly but washing/drying can alter SEI/CEI. ‐ ***Operando***: Preferred but challenging for vacuum techniques (XPS, SEM) due to liquid electrolyte volatility. In situ XPS with positive pressure is emerging but not widely implemented. **Protocols**: Rinsing is common but debated. **Challenges**: Not possible to characterize in *operando* under vacuum conditions.	‐ **In situ*/Operando***: Often necessary as components remain intact. Compatible with vacuum‐based techniques (*operando* XPS, SEM), but maintaining cell integrity and minimizing beam damage are crucial. (S)TEM, FIB/SEM with EDS, EELS, neutron reflectometry are used. Depth profiling or fracturing may be needed. **Challenges**: Difficult to separate components for ex situ analysis. Air sensitivity of some SEs (e.g., thiophosphates) requires careful handling.	‐ **In situ*/Operando***: Difficult due to polymer sensitivity to vacuum (outgassing, morphological changes) and beam damage. Requires regulated temperature setups. In situ microscopy (ATR‐FTIR, near‐field FTIR) employed. ‐ **E*x situ***: Problematic due to the adhesive nature making damage‐free disassembly hard. XPS used ex situ on both sides of the interface. **Challenges**: Avoiding alteration during disassembly and characterization due to adhesiveness and sensitivity.

##### Toward a Standardized Protocol for Battery Research

Even taking into consideration the most widely used techniques in the characterization of interfaces in the liquid, solid‐state, or polymer‐based electrolyte systems, it is the lack of standardization that affects the way we understand them. Indeed, the lack of standardization protocols on battery research has been subject to critical analysis over the years. Interlaboratory studies, such as those by Ohno et al.^[^ ^110^ ^]^ and Puls et al.^[^ ^111^ ^]^ reveal significant variability in reported electrochemical performance metrics for thiophosphate‐based solid electrolytes and all‐solid‐state batteries, even when using identical materials. Further Round‐Robin experiments were carried out in the literature to discuss the dispersity of the results in the battery field among laboratories.^[^ ^112^, ^113^, ^114^ ^]^

To promote transparency and reproducibility in battery research across academic and industrial environments, it is essential to comprehensively report parameters that have been identified as critical in research articles investigating the electrochemical performance of battery materials. Building on recommendations from recent literature,^[^ ^12^ ^]^ we strongly encourage authors to provide as much as possible of the information on materials, synthesis, cell fabrication, and practical experimental details contained in **Table** **3**:

**Table 3:** Parameters and sampling conditions that should be reported in papers to ensure a fair comparison between studies.

Material and Electrode Specification	
Common for each component	Specify the type, purity, supplier, and ideally batch/lot number.
Active Material	Provide morphology (e.g., SEM), particle size distribution, and specific surface area (e.g., BET analysis).
Conductive Additive	Report weight fraction (wt%), mixing method (equipment, duration, speed, volume ratios), surface analysis.
Binder	Include weight fraction (wt%), and solvent used for slurry preparation including solvent purity (water content as an example).
Current Collector	Provide the material (e.g., Al, Cu), thickness, and any surface pre‐treatment (e.g., mechanical, chemical etching).
Electrode Formulation	State the ratio of active material: conductive additive:binder and describe the slurry preparation protocol (mixing sequence, viscosity control, and degassing steps).
Electrode Properties	Include electrode mass loading (mg/cm^2^), thickness (µm, specify measurement method), and porosity (%, specify method). Describe the pristine surface state (e.g., as characterized by XPS or ToF‐SIMS) and drying properties. Storage conditions prior to cell assembly should also be specified.
**Electrolyte and Cell Assembly**	
Electrolyte Composition	Report solvent(s) with water content (ppm), salt type and concentration, storage and drying procedures, and any additives (concentration, purpose, and supplier).
Separator	Specify type, thickness, porosity, and any pre‐treatment steps (e.g., drying, plasma and cleaning).
Electrodes	For counter and reference electrodes, provide material, thickness, supplier, and surface pre‐treatment details (e.g., polishing, cleaning).
Assembly Environment	Include used casing (coin cell, pouch, or specific setups), electrolyte volume (µL per cell or µL per mg of active material), wetting procedure, glovebox brand and conditions (O₂ and H₂O levels in ppm, temperature), and stack pressure applied in solid‐state or pouch cells (MPa or psi, and method of application).
**Electrochemical Cycling Protocol**	
Formation Protocol	Report the number of cycles, C‐rates, voltage limits, temperature, and rest periods used during cell conditioning.
Cycling Conditions	Provide specific parameters such as C‐rate, voltage window, total number of cycles, and the targeted state of charge (SOC) or state of health (SOH) for characterization.
Temperature Control	Specify the cycling temperature (°C) and control method (e.g., climate chamber, thermoelectric platform).
Open Circuit Voltage (OCV) Conditions	Indicate the OCV hold time before disassembly or characterization, and the temperature during this period.
Testing Equipment	Include the manufacturer and model of the potentiostat/cycler used.
**Post‐Cycling Sample Handling and Preparation**	
Rest Time	Report the duration the cell rested at OCV after the final cycle and before disassembly.
Disassembly Conditions	Describe glovebox brand, atmosphere (e.g., Ar or N₂), O₂ and H₂O levels (ppm), and temperature.
Rinsing/Washing Protocols	Include solvents used, volume, number of rinses, duration, agitation method, and scientific justification.
Drying Procedure	Report drying method (e.g., vacuum, inert gas), temperature, and duration.
Storage and Transfer	Describe post‐preparation storage conditions (atmosphere, temperature, duration) and transfer methods to analytical instruments (e.g., vacuum/inert gas holders, cryo‐transfer, ambient exposure time).
Cross‐Sectioning/Polishing	Specify technique (e.g., mechanical, ion milling, cryo‐FIB) and relevant parameters (e.g., beam energy, polishing duration).
**Analytical Instrumentation and Data Acquisition**	
Techniques Used	List all analytical techniques employed (e.g., XPS, SEM, TEM, ToF‐SIMS, and Raman), specifying their application focus.
Instrument Details	Provide the manufacturer and model for each technique.
Beam Sensitivity and Mitigation	Describe protocols to prevent beam‐induced damage (e.g., low‐dose imaging, cryogenic analysis, and test exposures).
Region of Interest	Define criteria for selecting analysis areas, and report area or volume size.
Key Acquisition Parameters	Include technique‐specific parameters (e.g., for XPS: X‐ray source, pass energy, take‐off angle; for ToF‐SIMS: primary ion type, dose, energy; for SEM/TEM: accelerating voltage, and detectors used).
Acquisition Metrics	Report data collection duration per spectrum/image and number of spots or areas analyzed to ensure statistical representativeness.
Calibration	Include instrument calibration procedures and reference standards used (e.g., binding energy standards for XPS).
**Data Processing and Validation**	
Software	Specify the name and version of software and if necessary, the package used for data analysis.
Processing Steps	Outline key processing methods and model used such as background subtraction, peak fitting parameters and constraints, filtering, and image correction techniques.
Reference Databases	Indicate databases or libraries used for spectral interpretation.
Quantification Method	Describe how quantitative values were derived, including any sensitivity factors or correction schemes applied.
Statistical Analysis	Report number of replicate measurements, error analysis approach, and assessment of reproducibility.
Comparison with Controls	Describe how cycled sample data were benchmarked against control or pristine (uncycled) samples.

##### The Role of Computational Modeling in Interface Studies

Although computational modeling and simulations are out of the scope of this review, they have become useful for gaining atomic‐level insights into the complex interfacial phenomena occurring in batteries; information that would be too difficult―if not impossible―to access experimentally. Computational modeling has emerged as a powerful complement to experiments for unraveling buried battery interface phenomena. First‐principles such as Density functional theory (DFT), classical molecular dynamics (MD) and *ab initio* MD provide atomic‐scale thermodynamic and kinetic insights, while machine‐learned force fields (e.g., deep potential molecular dynamics, DPMD) significantly extend accessible simulation time/length scales. For example, DPMD simulations of a Li|Li_6_PS_5_Cl solid‐electrolyte interface directly revealed Li‐cluster (incipient dendrite) nucleation inside the SEI region,^[^ ^115^ ^]^ and classical MD studies show that highly concentrated electrolytes restructure the electric double layer–excluding solvent from the electrode–to extend electrochemical stability.^[^ ^116^ ^]^ Recent reviews emphasize that these multiscale simulations (classical, *ab initio*, and machine learning‐based MD) are invaluable for probing electrolyte structure, SEI formation, and interfacial reactions that are otherwise difficult to access experimentally.^[^ ^116^, ^117^ ^]^ In particular, Nolan et al. note that computation can accelerate the design of new electrolyte materials and interfaces for solid‐state batteries^[^ ^118^ ^]^ illustrating how atomistic simulation insights synergize with experiment to guide discovery of more robust interfacial chemistries.

## 2 Conclusion

The detailed investigation of the interphase–the region between material layers in Li‐ion and other M‐ion batteries–is crucial for optimizing electrochemical performance. These interphases, composed of complex organic/inorganic decomposition products, are characterized by their extremely thin constitution and their high reactivity to air/moisture and to manipulation (cell dismantled, beam damage, etc.). The approach for investigating interphases depends, among other factors, on whether the electrolyte is liquid, solid, or hybrid/polymer‐based. Several characterization techniques such as XPS, X‐ray fluorescence (XRF), Raman spectroscopy, or Fourier‐transform infrared spectroscopy (FTIR) methods offer promising avenues for investigating them, but they also pose a risk of altering their physico‐chemical nature. Even with the advent of *operando* techniques, which offer a considerable advantage by providing a dynamic aspect of interphase formation, accessing reliable data remains challenging. This difficulty arises from the inherent complexity of these interphases, coupled with the notorious lack of standardized probing protocols. The need for rigorous sample preparation methods as well as the importance of the non‐alteration of the interfacial layers during analysis are key factors that influence not only the quality of the results, but their interpretation as well. Furthermore, the data available in the literature is highly scattered due to lack of standardization, making it difficult to compare results across studies. This inconsistency, driven by variations in methodology and probing criteria, hinders progress toward a deeper understanding and the development of new solutions. We must prioritize the design of standardized research protocols that include a detailed specification of measurement conditions. As a starting point, results should also be compared to the pristine stage electrode and one in contact with electrolyte without any electrochemical activities to separate the chemical decomposition from the electrochemical decomposition.

Additionally, the buried nature of certain interfaces, particularly in solid‐state batteries, presents additional obstacles. Therefore, new measurement strategies must be devised and the implementation of existing techniques, such as depth profiling and chemical deposition, must be improved to make these interfaces more accessible and comprehensible. Ultimately, a better understanding of these buried interfaces will drive significant improvements in battery electrochemical performance, cycle life, and safety, enabling them to meet the demanding requirements of applications such as electric mobility and large‐scale renewable energy storage.

The future of research in this field relies on stronger collaboration between scientific communities, the development of standardized methodologies, and the refinement of analytical tools. These advances will not only enhance our ability to characterize interfaces but will also support the design of more efficient and safer batteries, contributing to solutions for global energy challenges.

## Conflict of Interest

The authors declare no conflict of interest.
